# FGF signalling through Fgfr2 isoform IIIb regulates adrenal cortex development

**DOI:** 10.1016/j.mce.2013.01.014

**Published:** 2013-05-22

**Authors:** Leonardo Guasti, W.C. Candy Sze, Tristan McKay, Richard Grose, Peter J. King

**Affiliations:** a Centre for Endocrinology, William Harvey Research Institute, Queen Mary University of London, London, UK; b Tumour Biology, Barts Cancer Institute, Queen Mary University of London, London, UK

**Keywords:** Shh, sonic hedgehog, FGFR, fibroblast growth factor receptor, Adrenal, Development, FGF, FGFR2, Shh

## Abstract

Developmental signalling pathways are implicated in the formation and maintenance of the adrenal gland, but their roles are currently not well defined. In recent years it has emerged that Sonic hedgehog (Shh) and Wnt/β catenin signalling are crucial for the growth and development of the adrenal cortex. Here we demonstrate that Fibroblast growth factor receptor (Fgfr) 2 isoforms IIIb and IIIc are expressed mainly in the adrenal subcapsule during embryogenesis and that specific deletion of the Fgfr2 IIIb isoform impairs adrenal development, causing reduced adrenal growth and impaired expression of SF1 and steroidogenic enzymes. The hypoplastic adrenals also have thicker, disorganised capsules which retain *Gli1* expression but no longer express Dlk1. Fgfr2 ligands were detected in both the capsule and the cortex, suggesting the importance of signalling between the capsule and the cortex in adrenal development.

## Highlights

► The adrenal glands from *fgfr2IIIb* knock-out mice are hypoplastic. ► Steroidogenic enzymes are reduced in *fgfr2IIIb* knock-out mice. ► Dlk1, rather than Shh, signalling is affected in the adrenal cortex of *fgfr2IIIb* knock-out mice. ► FGF1 is proposed as the ligand for FGFR2IIIb in the adrenal cortex.

## 1 Introduction

The adrenal cortex is the primary site of steroid synthesis, producing glucocorticoids under the control of the hypothalamic–pituitary–adrenal (HPA) axis and mineralocorticoids under the control of the renin-angiotensin system (RAS). Adrenocortical development originates in a group of cells lying between the urogenital ridge and the dorsal aorta, forming the precursors of the adrenal glands and the gonads, the adrenogonadal primordium (AGP) (Hatano et al., 1996). At around embryonic day e9.0 in the mouse these cells begin to express the steroidogenic transcription factor Steroidogenic Factor-1 (SF1), which is essential for both adrenal and gonadal development (Luo et al., 1994). By e12.0, cells of the dorsal medial AGP form the adrenocortical primordium which is invaded by migrating sympathetic neural crest cells that form the central adrenal medulla (Hatano et al., 1996). The gland is also encapsulated at this time in a poorly understood manner. Zonation occurs around birth (for reviews see (Kim et al., 2009; Laufer et al., 2012). Over the last few years signalling pathways involved in adrenal development have begun to be elucidated. We and others have shown that sonic hedgehog (Shh) is expressed in relatively undifferentiated steroidogenic cells in the subcapsular region of the mouse adrenal from e12.5 throughout life (King et al., 2009; Ching and Vilain, 2009; Huang et al., 2010). The Shh signal is transduced by capsule cells, some of which delaminate into the cortex, lose their responsiveness to Shh, and become steroidogenic (King et al., 2009; Laufer et al., 2012). Deletion of *Shh* from steroidogenic tissues causes adrenal hypoplasia, and interestingly the adrenal capsule is markedly thinner. Similar targeted deletion of β *catenin*, which like Shh is expressed just underneath the capsule in the murine adrenal cortex from e12.5, also causes adrenal hypoplasia, demonstrating that canonical Wnt signalling is critical for adrenal development.

Like Shh and Wnts, the pathways activated by the binding of Fibroblast Growth Factors (FGFs) to their receptors are of crucial importance for many developmental processes as well as in disease (Ornitz and Itoh, 2001; Turner and Grose, 2010). Four signalling FGF receptors, FGFR1-4, have been identified; these are transmembrane tyrosine kinases which contain three extracellular immunoglobulin (Ig) domains. FGFRs 1–3 are alternatively spliced in the third Ig domain such that an invariant exon IIIa is spliced to either exon IIIb or IIIc to produce IIIb or IIIc isoforms. Embryos with a global *Fgfr2 IIIb* deletion have hypoplastic adrenals (Revest et al., 2001), and deletion of both isoforms of FGFR2 from steroidogenic tissue recapitulates this phenotype (Kim et al., 2007), implying that the receptor is both expressed in the adrenal cortex and necessary for development. However, neither the expression pattern of FGFR2 isoforms, nor the adrenal phenotypes of the knockouts, has been investigated further.

Here we demonstrate that Fgfr2 IIIb and IIIc isoforms are expressed underneath the embryonic adrenal capsule, in a similar expression pattern to Shh and β catenin. *Fgfr2 IIIb* null mouse embryos have hypoplastic adrenals with impaired steroidogenic differentiation, and a larger and less defined capsule. However, the expression of the Shh target gene *Gli1* in capsular cells persisted in *Fgfr2 IIIb* null adrenals. Instead, the capsular expression of *Dlk1* (also known as *Pref-1*), a member of the Notch family of EGF repeat-containing proteins (for review see (Sul, 2009)), was strongly and specifically reduced in the *Fgfr2 IIIb* knock-out. Finally, we performed RT-PCR analysis of FGFs in laser capture dissected mouse embryonic adrenal tissues, and detected *Fgf1* in the adrenal capsule and *Fgf2* and *Fgf9* in the adrenal cortex.

## 2 Methods

### 2.1 Mice

Mice were housed in rooms with controlled light and temperature and treated under the Home Office Animals (Scientific Procedures) Act 1986.

We used Cre-Lox transgenics to generate *Fgfr2 IIIb* null embryos, taking advantage of a K5^cre^ construct which, in female mice, is mis-expressed in the oocyte in addition to basal keratinocytes, resulting in global deletion of Fgfr2 IIIb (Grose et al., 2007; Ramirez et al., 2004). Briefly, *Fgfr2 IIIb ^flox/flox^* male mice were crossed with female *keratin K5^cre/+^*; *Fgfr2 IIIb ^flox/+^* mice. Wild type and *K5^cre/+^* females were sacrificed by CO_2_ asphyxiation at day 15.5 post-fertilisation and embryos were snap frozen in liquid nitrogen and stored at −80 °C. Sagittal sections were obtained on a freezing-microtome (Leica GM 1510 S or Leica 3050 S) at 12 μm thickness, and mounted on Superfrost Plus slides (VWR). Sections were stained with hematoxylin and eosin using standard procedures.

### 2.2 Non-radioactive *in situ* hybridization (NR-ISH)

RNA was extracted from adult adrenal tissues using an RNeasy Mini kit (Qiagen), and cDNA was prepared from random-primed total RNA using Maloney Murine Leukemia Virus-Reverse Transcriptase (MMLV-RT, Promega). Fgfr2, Fgfr2 IIIb, Fgfr2 IIIc and Dlk1 cDNAs were amplified (PCR primer sequences are listed in Supplemental) and cloned into pGEM T-easy vectors (Promega). The vectors were sequenced and linearised with the appropriate restriction enzymes. The mouse *Gli1* probe (King et al., 2009) and *Sf1* probe (Guasti et al., 2011) have been described previously.

Digoxigenin (DIG)-labelled cRNA probes synthesis and NR-ISH was performed as described (Guasti et al., 2011).

### 2.3 Immunostaining

Immunostaining of sections and image acquisition and processing was performed according to (Guasti et al., 2011). Anti-Cyp11b1 (1:20 dilution), anti-P450 Side Chain Cleavage (Scc, 1:1000 dilution, Millipore AB1244), anti-PCNA (F2, sc25280, Santa Cruz Biotechnology, 1:1000 dilution) and anti-Cleaved caspase 3 (Cell Signaling, D175, 1:500 dilution) were used in this study. For immunohistochemical comparative analysis (as well as for *Gli1*, *Sf1* and *Dlk1* NR-ISH), all steps from fixation and sectioning to staining and image acquisition were performed in parallel in wild-type and knock-out mice.

### 2.4 Laser capture microdissection

Sections of e15.5 mouse embryos were cut at 12 μm thickness and mounted onto polyethylene napthalate-membrane slides (Life Technologies). Sections were fixed and stained with cresyl violet using the LCM staining kit (Ambion) according to the manufacturer’s instructions. Sections were allowed to dry at room temperature for 5 min and immediately used for laser capture microdissection using a PALM MicroBeam (Zeiss). Adrenal capsule, adrenal cortex and pooled tissues (brain, liver, heart, skin, gut, and lungs) were collected in RLT lysis buffer (RNeasy kit, Qiagen) and RNA was extracted according to the manufacturer’s instructions. cDNA was synthesised using a Sensiscript RT kit (Qiagen) according to the manufacturer’s instructions.

## 3 Results

### 3.1 Expression pattern of FGFR2 isoforms

**Fig. 1:**
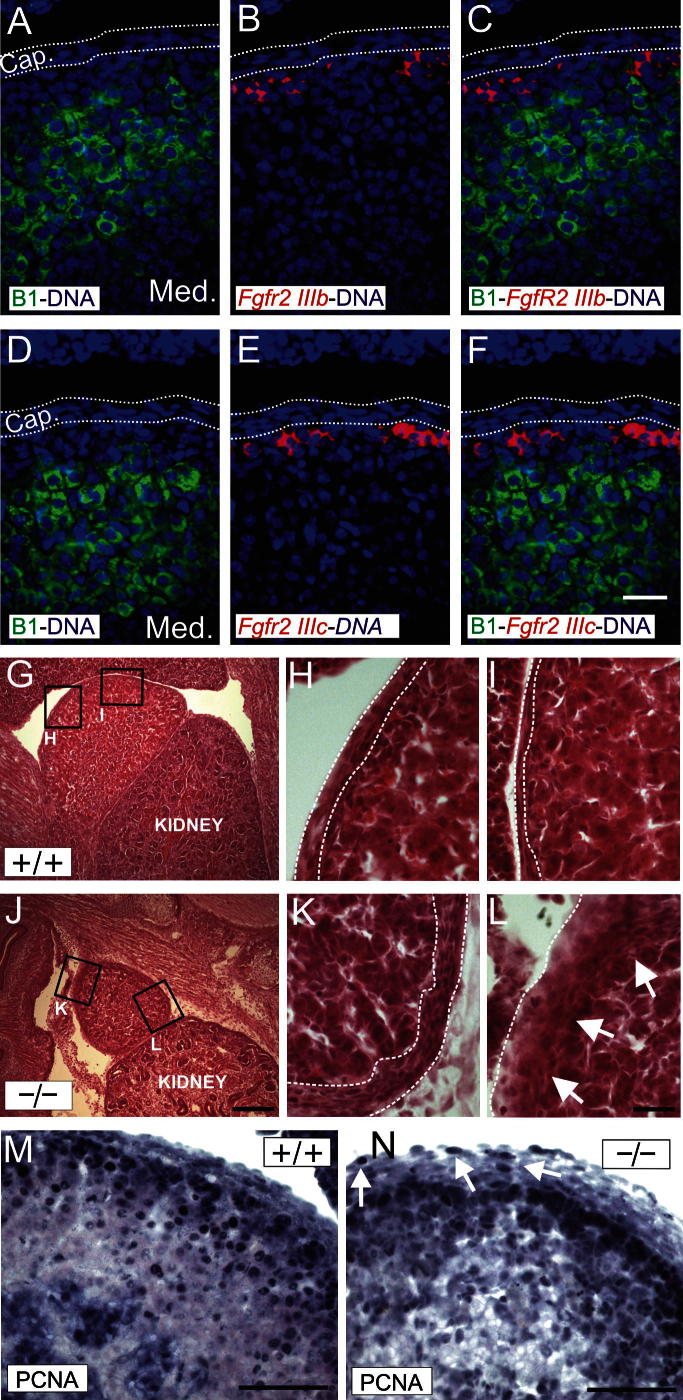
(A–F) NR-ISH analysis of e15.5 mouse adrenal glands using *Fgfr2 IIIb* (B and C) and *IIIc* (E and F) specific probes combined with Cyp11b1 immunolocalization. *Fgfr2* positive cells are labelled red, Cyp11b1 cells are labelled green and cell nuclei are labelled blue (Cap. = capsule, Med. = medulla). (G-L) Hematoxylin and eosin staining of wild-type (^+/+^, G–I) and *fgfr2 IIIb* knock out (^-/-^, J–L) adrenals. (M, N) Immunohistochemical analysis of PCNA expression in wild-type (^+/+^, M) and fgfr2 IIIb knock-out (^-/-^, N) adrenals. Arrows in N point to PCNA-positive cells in the adrenal capsule. Scale bars = F, 50 μm (applies to A–F); J, 150 μm (applies to G and J); L, 50 μm (applies to H, I, K, and L); M and N = 100 μm. (For interpretation of the references to colour in this figure legend, the reader is referred to the web version of this article.)

We investigated the expression of *Fgfr2* isoforms *IIIb* and *IIIc* in e15.5 wild type (WT) mouse adrenals by non-radioactive *in situ* hybridization (NR-ISH) using isoform-specific probes (Fig. 1A–F): both *IIIb* and *IIIc* isoforms were expressed underneath the capsule in cells that do not express Cyp11b1. This is the same expression pattern we observed for Shh in developing and adult mouse (King et al., 2009) and rat (Guasti et al., 2011) adrenals. A probe recognising both isoforms gave similar results; however, upon longer exposure, some capsular *Fgfr2* expression could be detected with this probe (Fig. S1).

### 3.2 *Fgfr2 IIIb* null embryos have hypoplastic adrenals

As *Fgfr2 IIIb* null embryos die at birth, we analysed e15.5 embryos. The adrenals in the null embryos are clearly smaller and have a markedly thicker capsule containing rounded rather than the typically elongated cells (Fig. 1G–L), and a less well-defined boundary with the cortex (arrows in Fig. 1L). The expression of *Fgfr2 IIIc* did not seem to be affected by *IIIb* deletion (Fig. S1). To investigate the mechanisms for the hypolasticity of the gland we studied markers of apoptosis and proliferation. While apoptotic cells (detected with an anti-cleaved caspase 3 antibody) were rare and mainly localised in the medulla in both WT and knock-out (KO) adrenals (Fig. S2), analysis of cell proliferation with an anti-PCNA antibody showed that, whereas the number of PCNA-positive cortical cells immediately adjacent to the capsule did not differ between WT and KO mice, PCNA positive nuclei were clearly present in the capsule of the KO, but absent in the WT, and there were fewer proliferative cells in the inner cortex of the KO adrenal compared to WT adrenals.

### 3.3 *Fgfr2 IIIb* null adrenals have reduced steroidogenic enzyme expression

**Fig. 2:**
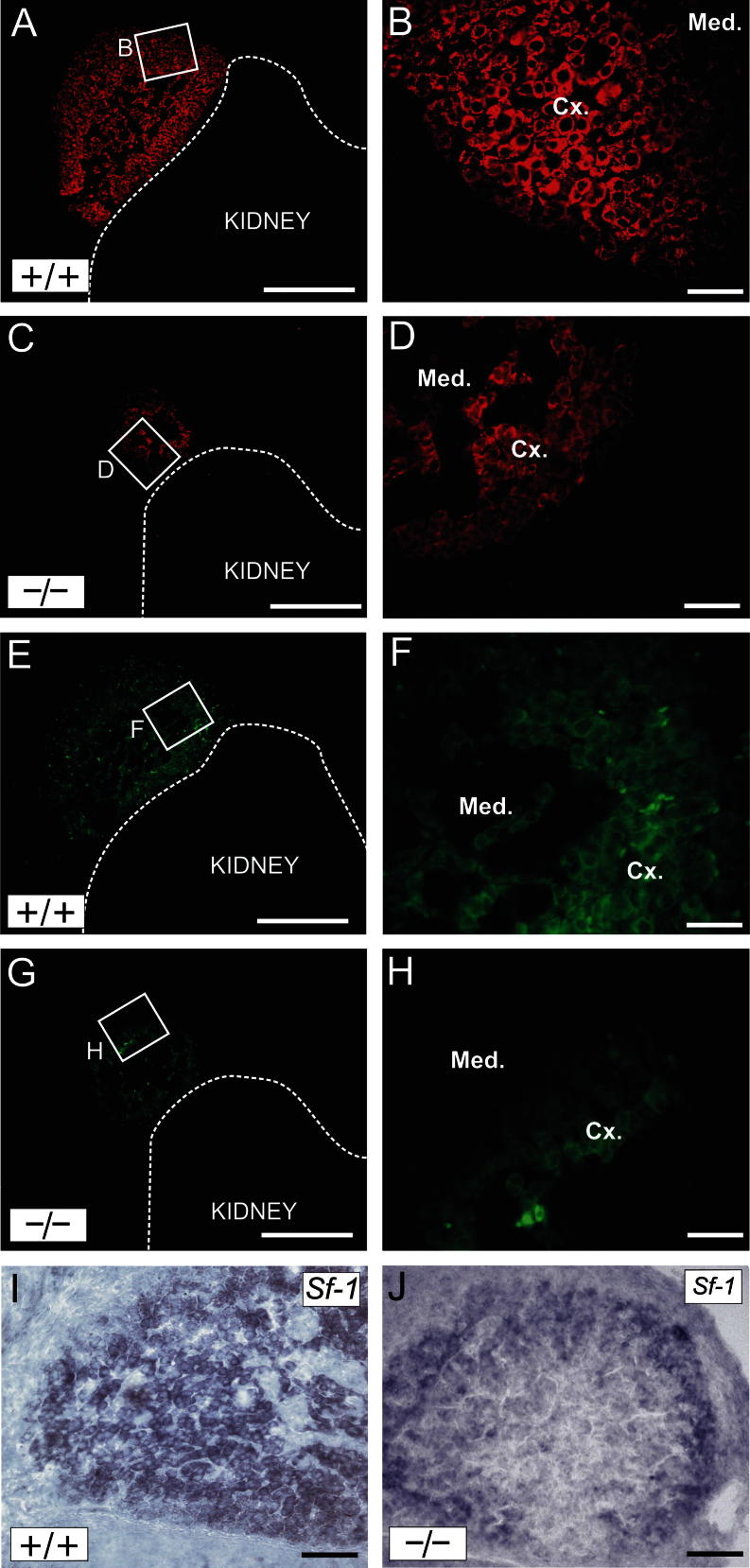
Expression of Scc (A–D), Cyp11b1 (E–H) and *Sf1* in e15.5 WT (A, B, E, F, and I) and *Fgfr2 IIIb* KO (C, D, G, H, and J) adrenals. Scale bar = 200 μm (A, C, E, and G); 50 μm (B, D, F, H, I, and J). Med. = medulla, Cx. = cortex.

Next, the expression of the pan-steroidogenic marker Cyp11a1 (Scc) and the ZF marker Cyp11b1 was compared between WT and knockout (KO) adrenals using specific antibodies (Fig. 2). The expression of both enzymes was noticeably reduced in KO embryos, with fewer cells expressing the enzymes and at a lower level. NR-ISH analysis also showed that *Sf1* mRNA expression was reduced in KO adrenals (Fig. 2I and J).

### 3.4 Analysis of gene expression in the capsule of *Fgfr2 IIIb* null adrenals

**Fig. 3:**
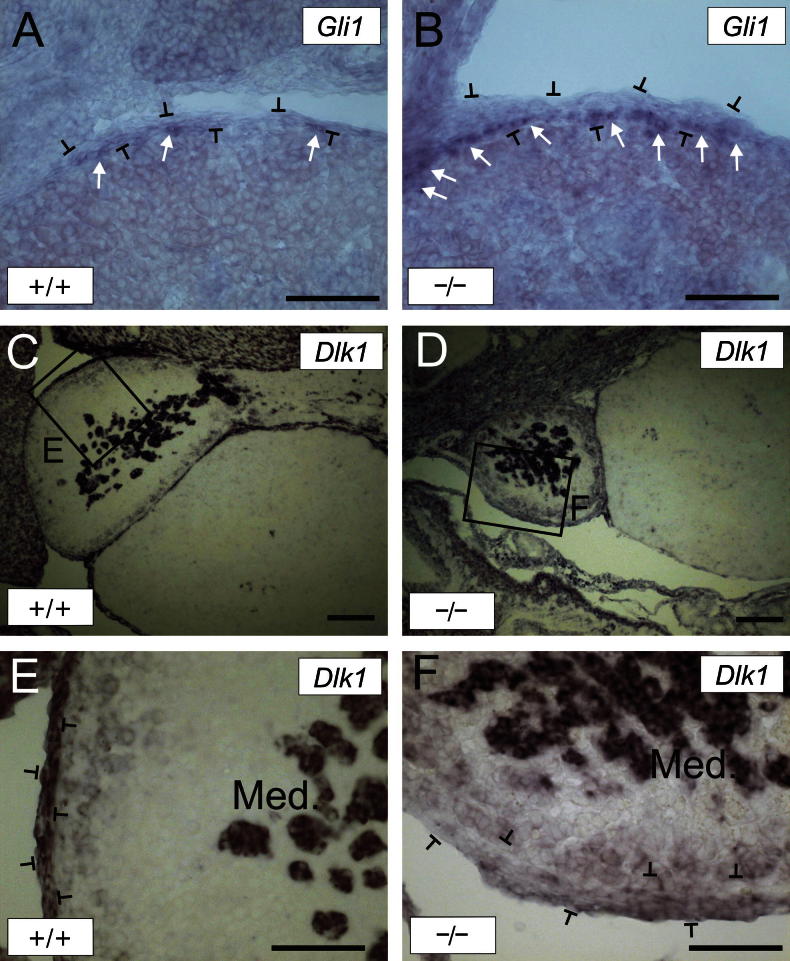
NR-ISH of WT (^+/+^) and *Fgfr2 IIIb* KO (^-/-^) e15.5 mouse adrenals using *Gli1* (A and B) and *Dlk1* (C–F) riboprobes. Arrows in A and B indicate clusters of *Gli1* positive cells in the capsule. Capsule cells in A, B, E and F are between the brackets. Scale bars = 100 μm. Med. = medulla.

As signalling through Fgfr2 IIIb is required to establish Shh expression in the developing limb bud (Revest et al., 2001), it was therefore possible that the defects in adrenal development described above are a consequence of perturbed Shh signalling. Shh activity at the capsule was assessed by *Gli1* NR-ISH (Fig. 3). *Gli1* expression was readily detected in the *Fgfr2 IIIb* KO adrenal capsule, and was apparently expressed at higher levels than in the wild type mouse, with more clusters of *Gli1* positive cells observed. To investigate the capsule phenotype further, we also studied the expression of *Dlk1*, another marker of the mouse adrenal capsule (Beuschlein et al., 2002), which is also expressed in the medulla. Interestingly, *Dlk1* expression was completely lost from the adrenal capsule but retained in the medulla, which is comparable in size to that of the wild type adrenal, indicating that medulla formation is unaffected.

### 3.5 Fgf expression in mouse embryo adrenals

**Fig. 4:**
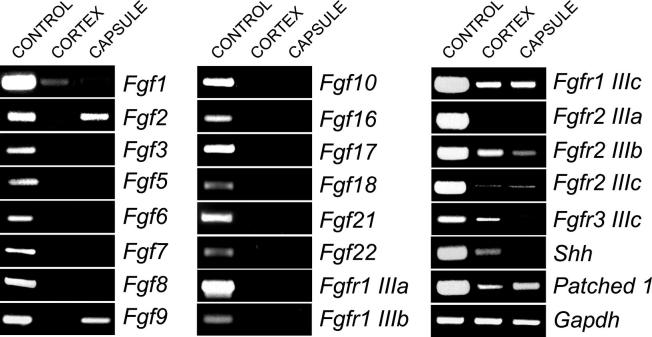
RT-PCR analysis of *Fgfs* and *Fgfrs* expression in laser capture microdissected e15.5 adrenal capsule and cortex. Control cDNA was made from pooled tissues including lung, heart, liver, bone and gut.

Finally, we performed laser capture microdissection on e15.5 WT mouse adrenal tissues, collecting tissues from both the capsule and the cortex. RT-PCR performed on RNA isolated from these tissues, using primers against FGF isoforms, detected expression of *Fgf1*, in the cortex, and *Fgf 2* and *9*, in the capsule (Fig. 4). The detection of *Shh* expression in the cortical fraction and the preferential expression of its receptor *Ptch1* in the capsule (King et al., 2009) indicated the accuracy of the microdissection. Both *Fgfr2 IIIb*, albeit at lower levels than in the cortex, and *IIIc*, were amplified in the capsule, in keeping with the NR-ISH experiments (see Fig. 1 and Fig. S1).

## 4 Discussion

Germline knockout of the *IIIb* exon of the *Fgfr2* gene results in mice that die at birth from multiple developmental defects, identifying Fgfr2 IIIb as a critical mediator of organogenesis (De Moerlooze et al., 2000; Revest et al., 2001). We have shown here that adrenal development is also affected, with a detectable but smaller gland at e15.5 in which expression of the steroidogenic markers Sf1, Scc and Cyp11b1 is reduced, compared to a wild type adrenal. Analysis of apoptosis and proliferation markers demonstrates that the hypoplasia is likely a consequence of a reduction of inner cortical cell proliferation. In *Shh* null adrenals, apoptosis and proliferation indices are largely normal in the cortex and the defect in growth is believed to be a consequence of the very thin capsule, from which mesenchymal cells differentiate to populate the steroidogenic cortex in the wild type organ. β *catenin* KO, however, does result in reduced cortical proliferation.

Fgf and Shh pathways interact in several developmental settings including the limb bud (Laufer et al., 1994), the cerebellum (Fogarty et al., 2007) and the lung (Chuang et al., 2003), and Shh has been shown to be under both positive and negative regulation by FGFs. In the developing limb, for example, Fgf10 signalling to the apical ectodermal ridge through Fgfr2 IIIb is required to initiate Shh expression in the zone of polarising activity in the posterior limb bud and thus establish the anterior–posterior limb axis (Revest et al., 2001), and expression of Shh is restricted to the posterior limb bud by inhibitory Fgf signalling in the anterior limb to control limb patterning (Mao et al., 2009; Zhang et al., 2009). The maintained expression of the Shh target gene *Gli1* that we observe in the adrenal capsule demonstrates that FGF signalling through Fgfr2 IIIb is not required for Shh signalling in this setting, and may act to restrict it, given the apparent increased *Gli1* expression in the *Fgfr2 IIIb* KO adrenal capsule. Fgf and Wnt/β catenin pathways also interact, with Fgf10 activating β catenin signalling in developing skin (Reddy et al., 2001), mammary tissue (Veltmaat et al., 2006) and liver (Berg et al., 2007; Mavila et al., 2012), and Fgfs 1, 4 and 8 activating β catenin signalling in murine embryonic liver (Sekhon et al., 2004). We have not examined β catenin expression in the null adrenal but note that the phenotypes of the Fgfr2 IIIb and β catenin KO adrenals are similar, with impaired development, reduced expression of steroidogenic markers and reduced cortical cell proliferation evident (Kim et al., 2008). However, it does not appear that β *catenin* KO causes a capsule phenotype as we see here, so we do not expect that this aspect of the phenotype is a consequence of impaired Wnt signalling.

The two receptor isoforms are apparently predominantly expressed in the same cells in the cortex, although expression was detected in the adrenal capsule at lower levels. As the adrenal gland is present, signalling through Fgfr2 IIIb is not necessary for the formation of the adrenal primordium but, like Shh and β catenin (Kim et al., 2008), this receptor is required for the subsequent growth of the gland. The *Fgfr2 IIIb* KO examined here is a global knockout and it is therefore possible that the observed effects on adrenal development may be secondary to those on other organ systems, although no effect on the gonads was observed, indicating that the adrenal hypoplasia was not secondary to effects on the adrenogonadal primordium (Revest et al., 2001). Conditional deletion of both isoforms of Fgfr2 from steroidogenic tissue also leads to adrenal hypoplasia (Kim et al., 2007) and global hemizygous deletion of exon *IIIc* of *Fgfr2* does not appear to have any gross effect on adrenal development, unlike on the development of other visceral organs (Hajihosseini et al., 2001). Taken together, these observations suggest that the effect of loss of Fgfr2 IIIb on the adrenal is direct, although formal proof would require the generation of adrenal specific knockouts.

Fgfr2 IIIb is activated by five known ligands, Fgfs 1, 3, 7, 10 and 22 (Zhang et al., 2006), but of these we could only detect *Fgf1*, which is expressed in the cortex. *Fgfr2 IIIc* is also expressed in the adrenal cortex, and Fgfs 2 and 9 (Lavine et al., 2005) activate this isoform, both being detected in the capsule. Fgf2 acts as a mitogen for adrenocortical cells in culture and in regeneration experiments (Crickard et al., 1981; Lepique et al., 2004; Chu et al., 2009). However, the normal adrenal phenotype observed on hemizygous *Fgfr2 IIIc* deletion suggests that signalling through this receptor may not be necessary during development (Hajihosseini et al., 2001). Given the detection, albeit at low levels, of receptor expression in the adrenal capsule, it is not possible to determine whether Fgf1 is acting on the IIIb isoform receptor in the cortex or in the capsule, or both. It will be interesting to determine the relative importance of signalling from the cortex to the capsule (Fgf1/Fgfr2 IIIb), from the capsule to the cortex (Fgfs 2 and 9/Fgfr2 IIIc) or within the capsule (Fgfs 2 and 9/Fgfr2 IIIc) or cortex (Fgf1/Fgfr2 IIIb), for adrenal growth and development.

The thicker and disorganised adrenal capsule phenotype we observe is similar to that seen in *melanocortin 2 receptor (Mc2r*, *ACTH receptor)* KO adrenals (Chida et al., 2007), perhaps indicating an interaction between the FGF and ACTH signalling pathways, and this warrants further investigation. However, as both *Mc2r* and *Pomc* (Karpac et al., 2005) KO adrenals are otherwise normal at birth, we do not feel that impaired ACTH signalling is a likely mechanism for the effects on the growth and differentiation of the gland we see here. The observation that *Dlk1* expression is specifically lost from the adrenal capsule in the null adrenals is fascinating and suggests that this may be the cause of the capsule phenotype. Enucleation experiments in rats, in which the inner mass of the adrenal is removed and the cortex regenerates from the capsule and adherent subcapsular cells, have demonstrated that Dlk1 expression is immediately downregulated and not re-established until the zonation of the cortex has been restored (Halder et al., 1998). This led to the suggestion that Dlk1 is a negative regulator of adrenal differentiation, analagous to its role in adipogenesis (Smas and Sul, 1993). Unusually, however, the expression pattern of Dlk1 differs between rats and mice, with it being specifically expressed in subcapsular cells in the rat (Halder et al., 1998), rather than in the capsule (Beuschlein et al., 2002), perhaps making comparisons of the role of the protein in the two species invalid. Nevertheless, it is possible that Dlk1 negatively regulates differentiation of the Gli1-positive capsular stem cells in a similar manner and its downregulation is in response to the impaired recruitment of these cells into the cortex caused by the *Fgfr2 IIIb* KO, perhaps as a consequence of the effects on the growth and organisation of the capsule.

These data highlight the importance of FGF signalling in adrenal development. Shh expression is not greatly perturbed by the loss of FGF signalling, so presumably other, as yet unidentified, mechanisms are involved in the control of adrenal development and growth of the capsule by signalling through Fgfr2 IIIb. Determining these mechanisms is an important goal for understanding adrenal development.
