# Supplementary material for: FGF signalling through Fgfr2 isoform IIIb regulates adrenal cortex development
Source: Mol Cell Endocrinol. 2013 May 22;371(1-2):182–8. doi: 10.1016/j.mce.2013.01.014 (PMC3650577; doi:10.1016/j.mce.2013.01.014)
Supplement: Supplementary data 1 [file mmc1.docx]

**Supplementary data**

**RT-PCR primers**

mFGF1 F 5’-GGGGCCACTTCTTGAGGAT-3’

mFGF1 R 5’-GGTTTTCTTCCAGCCTTTCC-3’

mFGF2 F 5’-ACCAGGCCACTTCAAGGAC-3’

mFGF2 R 5’-GCCGTCCATCTTCCTTCATA-3’

mFGF3 F 5’-GCAAGCTCTACTGCGCTACC-3’

mFGF3 R 5’-TGCGTTGTAGTGATCCGAAG-3’

mFGF5 F 5’-TGTACTGCAGAGTGGGCATC-3’

mFGF5 R 5’-CACTCTCGGCCTGTCTTTTC-3’

mFGF6 F 5’-GGAGAGATTTCGGGTGTGAA-3’

mFGF6 R 5’-TCATGGCAATGAAGAGAGCA-3’

mFGF7 F 5’-GAACAAAAGTCAAGGAGCAACC-3’

mFGF7 R 5’-GTCATGGGCCTCCTCCTATT-3’

mFGF8 F 5’-GGAACCCAGCTGACACTCTC-3’

mFGF8 R 5’-CGGCTGTAGAGCTGGTAGGT-3’

mFGF9 F 5’-TTCCCCAACGGTACTATCCA-3’

mFGF9 R 5’-GTATCTCCTTCCGGTGTCCA-3’

mFGF10 F 5’-GAGAAGAACGGCAAGGTCAG-3’

mFGF10 R 5’-CTCTCCTGGGAGCTCCTTTT-3’

mFGF16 F 5’-CTGATCAGCATCAGGGGAGT-3’

mFGF16 R 5’-GGGTGAGCCGTCTTTATTCA-3’

mFGF17 F 5’-ACCCTGTGCTTGCAGCTATT-3’

mFGF17 R 5’-TCTGCTGCCGAATGTATCTG-3’

mFGF18 F 5’-CCTGCACTTGCCTGTGTTTA-3’

mFGF18 R 5’-CCCGAAGGTATCTGTCTCCA-3’

mFGF21 F 5’-ACCTGGAGATCAGGGAGGAT-3’

mFGF21 R 5’-GTCCTCCAGCAGCAGTTCTC-3’

mFGF22 F 5’-ACTTTTTCCTGCGTGTGGAC-3’

mFGF22 R 5’-TTGTAGCCGTTCTCCTCGAT-3’

mFGFR1 IIIa F 5’-AAAGCACATCGAGGTGAACG-3’

mFGFR1 IIIa R 5’-TTCATGGATGCACTGGAGTC-3’

mFGFR1 IIIb F 5’-TTAATAGCTCGGATGCGGAG-3’

mFGFR1 IIIb R 5’-ACGCAGACTGGTTAGCTTCA-3’

mFGFR1 IIIc F 5’-TGCTGGAGTTAATACCACCG-3’

mFGFR1 IIIc R 5’-CCAGAACGGTCAACCATGCA-3’

mFGFR2 IIIa F 5’-AAGGTTTACAGCGATGCCCA -3’

mFGFR2 IIIa R 5’-CTGCTGAAGTCTGGCTTCTT-3’

mFGFR2 IIIb F 5’-AAGGTTTACAGCGATGCCCA-3’

mFGFR2 IIIb R 5’-AGAGCCAGCACTTCTGCATT-3’

mFGFR2 IIIc F 5’-GTGTTAACACCACGGACAAA-3’

mFGFR2 IIIc R 5’-TGGCAGAACTGTCAACCATG-3’

mFGFR3 IIIc F 5’-GAGTTCCACTGCAAGGTGTA-3’

mFGFR3 IIIc R 5’-AGAACCTCTAGCTCCTTGTC-3’

mSHH F 5’-GATGAGGAAAACACTGGAGC-3’

mSHH R 5’-CCTCATAGTGTAGAGACTCC-3’

mPATCH1 F 5’-GCAGAGGACTTACGTGGAGG-3’

mPATCH1 R 5’-CTGACAGTGCAACCAACAGG-3’

mGAPDH F 5’- TGCACCACCAACTGCTTAG-3’

mGAPDH R 5’- GGATGCAGGGATGATGTTC-3’

**Primers for NR-ISH probes:**

Common region of FGFR2:

mFGFR2 F 5’-CAGAGGATCCAAAGTGGGAA-3’

mFGFR2 R 5’-AAACACAGAATCGTCCCCTG-3’

mFGFR2IIIb and mFGFR2IIIc specific primers:

mFGFR2IIIb/c F 5’-TGGGCTGCCCTACCTCAAGG-3’

mFGFR2 IIIb R 5’-CTTGCTGTTTGGGCAGGACA-3’

mFGFR2 IIIc R 5’-GAGTGAAAGGATATCCCG -3’

mDLK1 F 5’-TTCGGCCACAGCACCTAT-3’

mDLK1 R 5’-TATCCTCATCACCAGCCTCC-3’


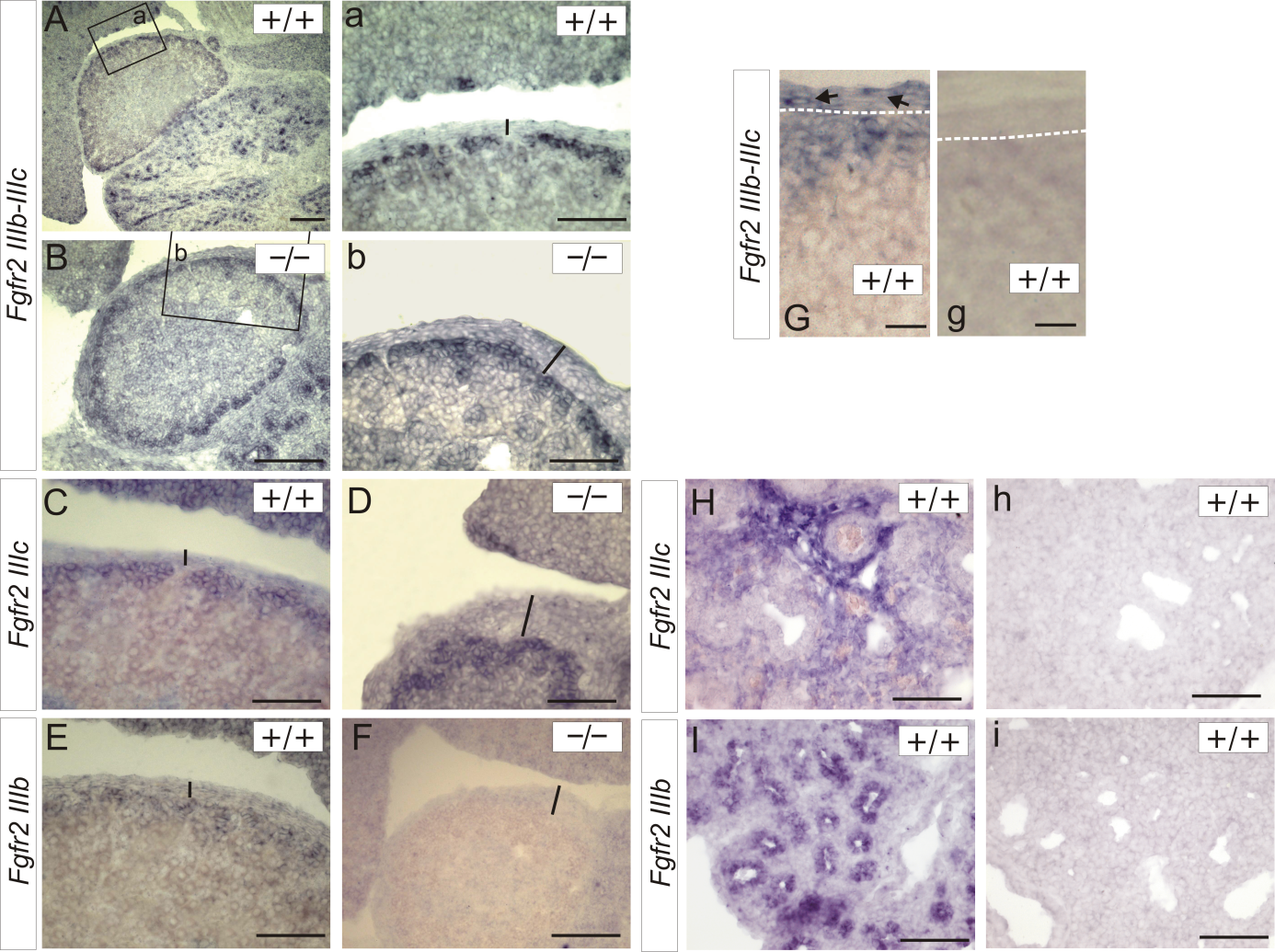


**Fig S1**. **NR-ISH of WT (+/+) and *fgfR2 IIIb* KO (-/-) e15.5 mouse embryos using a DIG-labeled cRNA antisense riboprobe recognizing a common mRNA region of FGFR2 (*fgfR2 IIIb-IIIc,* A, a, B, b, G, g) and riboprobes specific for *IIIc* (C, D, H) and *IIIb* (E, F, I) isoforms. Bars in the capsule indicate its thickness. Note the absence of specific signal in the KO adrenal when employing the *IIIb* specific probe (F). Positive cells can be observed in the subcapsular region with all probes, although some positive cells with a clear capsular localization could be detected only with the longer, common probe, and when exposing sections for longer (arrows in G). Sense probe resulted in no specific signal (g); the dotted lines in G and g indicate the capsule boundary.**

**The specificity of isoform specific-probes was further assessed in extra adrenal tissues where the differential expression of *IIIb* vs *IIIc* has been described: one such structure is the lung where the *IIIc* isoform was found to be predominant in the mesenchyme (H) while the *IIIb* isoform was mainly localized in bronchial epithelium (I), as described (De Moerlooze L. et al., 2000). Sense probes staining resulted in no specific signal (h and i). Scale bars = 100 μm, except G and g = 20 μm.**

[**De Moerlooze L**](http://www.ncbi.nlm.nih.gov/pubmed?term=De%20Moerlooze%20L%5BAuthor%5D&cauthor=true&cauthor_uid=10631169)**,**[**Spencer-Dene B**](http://www.ncbi.nlm.nih.gov/pubmed?term=Spencer-Dene%20B%5BAuthor%5D&cauthor=true&cauthor_uid=10631169)**, [Revest JM](http://www.ncbi.nlm.nih.gov/pubmed?term=Revest%20JM%5BAuthor%5D&cauthor=true&cauthor_uid=10631169), [Hajihosseini M](http://www.ncbi.nlm.nih.gov/pubmed?term=Hajihosseini%20M%5BAuthor%5D&cauthor=true&cauthor_uid=10631169), [Rosewell I](http://www.ncbi.nlm.nih.gov/pubmed?term=Rosewell%20I%5BAuthor%5D&cauthor=true&cauthor_uid=10631169),**[**Dickson C**](http://www.ncbi.nlm.nih.gov/pubmed?term=Dickson%20C%5BAuthor%5D&cauthor=true&cauthor_uid=10631169)**. An important role for the IIIb isoform of fibroblast growth factor receptor 2 (FGFR2) in mesenchymal-epithelialsignalling during mouse organogenesis.** [**Development.**](http://www.ncbi.nlm.nih.gov/pubmed/10631169)**2000 127: 483-492.**


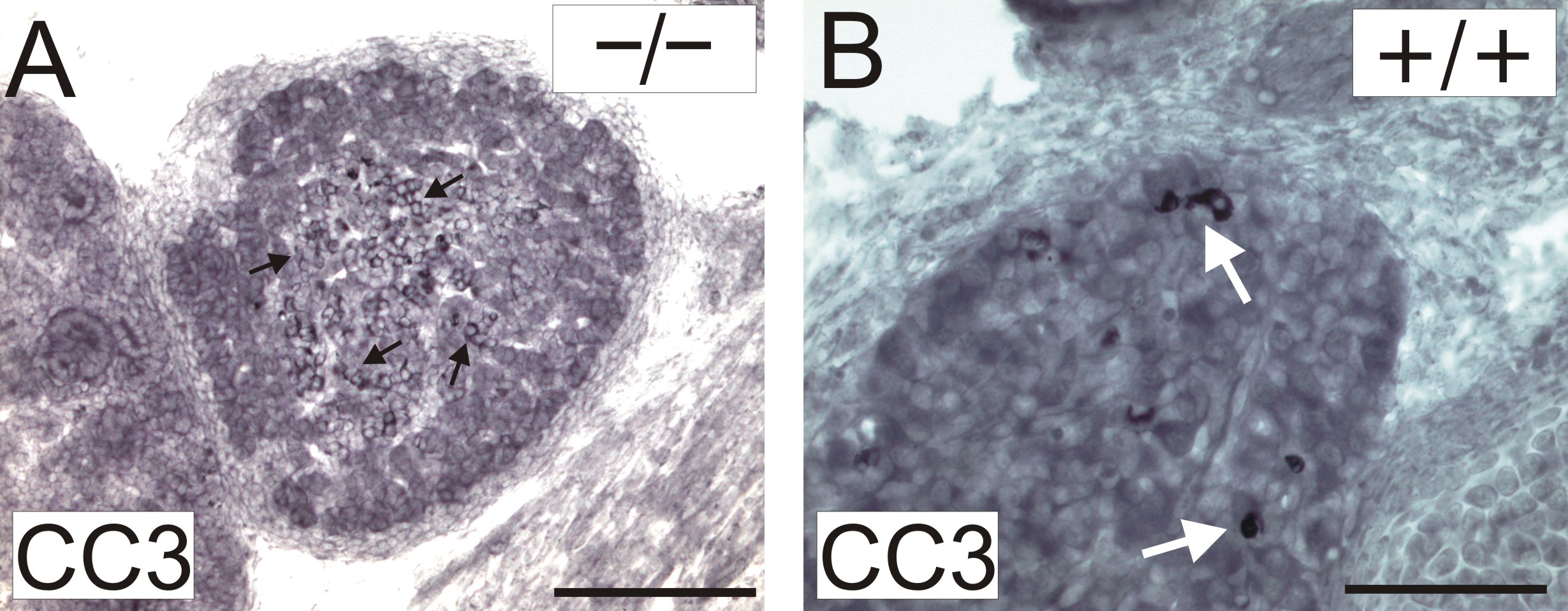


**Fig S2**. **Immunohistochemical analysis of cleaved-caspase 3 (CC3) positive cells in *fgfR2 IIIb* KO (A, -/-) adrenal gland and WT (D, +/+) dorsal root ganglia. Black arrows point to medullary cells. CC3 staining pattern was the same in WT adrenals (not shown). Strongly-labeled CC3-positive cells in dorsal root ganglia (arrows in D) are in keeping with published data indicating the specificity of the anti-CC3 antibody (Usui N. et al., 2012). Scale bars = 100 μm.**

[**Usui N**](http://www.ncbi.nlm.nih.gov/pubmed?term=Usui%20N%5BAuthor%5D&cauthor=true&cauthor_uid=22318233)**,**[**Watanabe K**](http://www.ncbi.nlm.nih.gov/pubmed?term=Watanabe%20K%5BAuthor%5D&cauthor=true&cauthor_uid=22318233)**,**[**Ono K**](http://www.ncbi.nlm.nih.gov/pubmed?term=Ono%20K%5BAuthor%5D&cauthor=true&cauthor_uid=22318233)**,**[**Tomita K**](http://www.ncbi.nlm.nih.gov/pubmed?term=Tomita%20K%5BAuthor%5D&cauthor=true&cauthor_uid=22318233)**, [Tamamaki N](http://www.ncbi.nlm.nih.gov/pubmed?term=Tamamaki%20N%5BAuthor%5D&cauthor=true&cauthor_uid=22318233), [Ikenaka K](http://www.ncbi.nlm.nih.gov/pubmed?term=Ikenaka%20K%5BAuthor%5D&cauthor=true&cauthor_uid=22318233), [Takebayashi H](http://www.ncbi.nlm.nih.gov/pubmed?term=Takebayashi%20H%5BAuthor%5D&cauthor=true&cauthor_uid=22318233). Role of motoneuron-derived neurotrophin 3 in survival and axonal projection of sensory neurons during neural circuit formation.** [**Development.**](http://www.ncbi.nlm.nih.gov/pubmed/22318233)**2012. 139:1125-1132**
